# First Meeting of the London Regional Committee

**Published:** 1921-01-08

**Authors:** 


### THE BRITISH HOSPITALS ASSOCIATION.

### First Meeting of the London Regional Committee.

The first meeting of the London Regional Committee of the British Hospitals Association was held at 19 Berkeley Street, W. 1, on Friday, December 17, 1920. There were present: The Right Honourable Viscount Hambleden (King's College Hospital), in the chair; the Marquess of Northampton, Great Northern Hospital; Earl Winterton, M.P., chairman, Hospital for Women, Soho; Viscount Goschen, Guy's Hospital; Lord Muir Mackenzie, Fever Hospital and others of group; Sir William Lawrence, Bart., St. Bartholomew's Hospital; Sir Ernest Hatch, University College; Sir Edward S. Hope, Hospital for Sick Children, Great Ormond Street; Sir Edward Penton, K.B.E., Middlesex Hospital; Sir Charles Russell, Hospital of St. John and St. Elizabeth; Sir Samuel Scott, Queen Charlotte's Hospital; Mr. R. H. Caird, London Homœopathic Hospital; Mr. A. St. George Caulfeild, D.L., J.P., Cancer Hospital (Free); Miss B. M. Davidson, South London Hospital for Women; Mr. Montague Ellis, Royal Free Hospital; Commander T. J. Farrell, London Fever Hospital; Mr. F. J. Frankau, St. George's; Mr. A. Lister Harrison, J.P., Metropolitan Hospital; Mr. F. L. Jermyn, King Edward Memorial, Ealing; Mr. T. W. Luling, Royal London Ophthalmic Hospital; Mr. C. E. Leo Lyle, M.P., chairman, Queen Mary's Hospital for the East End; Mr. E. Vernon Miles, Hampstead General Hospital; Mr. A. Morley, O.B.E., Royal National Orthopædic Hospital; Mr. E. W. Morris, C.B.E., London Hospital, E. 1; Lt.-Col. W. Parkes, D.S.O., St. Mary's Hospital, Paddington; Mr. Jno. A. Ritchie, Hornsey Cottage Hospital; Mr. Charles Stone, Miller General Hospital, London, S.E.; Mr. Austin Taylor, Westminster Hospital; Mr. L. A. Wallace, St. Thomas's Hospital; Mr. H. E. West, Royal National Orthopædic Hospital; Lt.-Col. E. M. Wilson, D.S.O., Cheyne Hospital for Children, Chelsea.

The President, Sir Arthur Stanley, shortly explained the objects of the newly constituted Regional Committee, and moved that Lord Hambleden be elected chairman.

The following letter from King Edward's Hospital Fund for London was read:

December 9, 1920.

The Hon. Sir Arthur Stanley, G.B.E.,
President, British Hospitals Association.

Dear Sir,—The Executive Committee of the King's Fund have given their most earnest consideration, at several recent meetings of the Committee, to the pressing problem of the financial difficulties of the London Voluntary Hospitals.

In connection with possible remedies they have discussed the following:—

(*a*) The suggestions made by the Fund in the circular letter sent with the emergency grants on July 5 last, that the hospitals should consider the question of payments from patients able to pay and systematic collections from employers and employed; and the replies received from the hospitals down to the end of November.

(*b*) The suggestion that public authorities should in future pay the full cost of patients for whom these authorities have taken responsibility; full cost including a proper share of overhead charges, interest on capital, depreciation, etc.; that such patients should include; (1) tuberculosis patients; (2) venereal patients; (3) maternity patients; (4) pensioners; (5) school children; (6) insured persons; (7) other cases, if any; and that in some cases the difference between such full payment and the amount actually paid should be made up retrospectively, *e.g.*, naval or military patients.

(*c*) The suggestion that a carefully guarded provision might be made for the exemption of voluntary contributions from assessment to income tax.

The Executive Committee have decided to recommend that, in the absence of any conclusive reason to the contrary, all these suggestions should be adopted as expressing the policy to be advocated by the King's Fund. They have already appointed a sub-committee to make a thorough examination of suggestion (*c*), and, if satisfied that the proposal is practicable, to prepare a draft of a scheme for the consideration of the authorities.

In the meantime they have directed me to communicate these facts to the Committee of the British Hospitals Association and to say that they would welcome any observations which the Association might wish to make on the subject.—Yours faithfully,

(Signed) H. R. Maynard, *Secretary.*

In regard to Section (*a*) of the letter, after some discussion, in which it was suggested that, if "collections from employers and employed" were to be generally made by individual hospitals, it would be necessary to allot a district to each hospital—it was decided to take no action.

In regard to Section (*b*), on the motion of Lord Goschen, seconded by Sir Edward Penton, it was resolved (1) That Sir Arthur Stanley approach the King's Fund with a view to arranging that the collection of the information required be carried out by the London Regional Committee; and (2) that, with a view to ascertaining what financial effect payments for the services detailed in the letter from the King's Fund would have in relieving the hospitals' present financial difficulty, a Sub-Committee be formed, consisting of Lord Hambleden, Sir Arthur Stanley, with Sir Napier Burnett, to obtain the information asked by the King's Fund in their letter of December 9, tabulate the same, and report, with recommendations, to the London Regional Committee.

In regard to Section (*c*), it was agreed that the matter of obtaining exemption from assessment to income tax on charitable contributions was a question that should be left to the King's Fund.

Letters were read from Lord Cheylesmore (chairman, Brompton Hospital for Consumption) and from Sir E. Kaye Butterworth (chairman of the City of London Hospital for Diseases of the Chest), asking for additional representation on the London Regional Committee. The matter was left to the Chairman, who was empowered to act as he deemed best.

A letter was read from the Queen's Hospital for Children withdrawing from the Association on the ground of the action taken at the Conference held on December 2 in regard to the representation of children's hospitals. Which also was duly noted.

A message from Lord Knutsford was read pointing out, with reference to the representation of the London Hospital by Mr. E. W. Morris, the House-Governor, in the event of his Lordship being unable to attend, that no decision had been made on December 2 at the meeting held at the Barnes Hall of the Royal Society of Medicine, definitely precluding the nomination of salaried officers to the London Regional Committee. Which was agreed.

A letter from the Hospital Saturday Fund addressed to hospitals was read. It was agreed that in future joint action in answer to such letters was advisable; but it was understood that in the present case answers had been already addressed to the Hospital Saturday Fund by various hospitals, and that no joint action was possible.

The date of the next meeting of the London Regional Committee was fixed for Tuesday, January 11, 1921, at half-past three o'clock in the afternoon, at 19 Berkeley Street, W.

